# Nonlinear and Vessel-Specific Association Between Coronary Artery Calcium Burden and Coronary Computed Tomography Angiography Underestimation of Coronary Stenosis

**DOI:** 10.31083/RCM52230

**Published:** 2026-09-08

**Authors:** Xuzhe Wang, Chengzhuo Wang, Yu Xie, Wenyu Wang, Ruifeng Liu

**Affiliations:** ^1^Department of Cardiology, Beijing Friendship Hospital, Capital Medical University, 100050 Beijing, China

**Keywords:** coronary computed tomography angiography, coronary artery calcium, diagnostic accuracy, stenosis underestimation, blooming artifact, generalized estimating equations

## Abstract

**Background:**

Coronary artery calcium (CAC) introduces substantial artifacts in coronary computed tomography angiography (CCTA). Although calcification is classically associated with overestimation of stenosis severity, underestimation may be even more clinically hazardous because underestimation can lead to inappropriate deferral of revascularization. Thus, this study aimed to investigate the association between CAC burden and CCTA underestimation, with emphasis on vessel-specific anatomical heterogeneity.

**Methods:**

We retrospectively evaluated 673 patients without known coronary artery disease who underwent both CCTA and invasive coronary angiography (ICA) within 90 days, yielding 2692 vessel observations. Underestimation was defined strictly at the vessel level as a CCTA stenosis grade lower than the corresponding ICA grade. Restricted cubic splines (RCS) were used to characterize the nonlinear association between CAC burden and the risk of underestimation. Within-patient clustering across the left main (LM), left anterior descending (LAD), left circumflex (LCX), and right coronary artery (RCA) was accounted for using generalized estimating equations (GEEs).

**Results:**

Any-vessel underestimation occurred in 389 patients (57.8%). Vessel-specific susceptibility varied markedly: LM, 3.0%; LAD, 27.9%; LCX, 29.3%; and RCA, 22.4%. RCS analysis demonstrated a significant nonlinear association between total CAC score and patient-level underestimation in the fully adjusted model (*p*-overall = 0.014; *p*-nonlinear = 0.004), with an upward risk trajectory observed as calcification increased relative to the median reference of 301. GEE modeling confirmed that patient-level CAC burden independently predicted vessel-level underestimation (CAC ≥400 vs. 0: odds ratio, 2.01; 95% confidence interval, 1.20–3.36; *p* = 0.008), with a significant interaction between CAC and vessel territory (*p* < 0.001). A sensitivity analysis restricted to patients without any CCTA overestimation (n = 155) confirmed a robust nonlinear association between total CAC score and pure underestimation risk (*p*-overall = 0.012; *p*-nonlinear = 0.005), suggesting that concurrent overestimation noise partially obscures this dynamic risk trajectory in the full cohort.

**Conclusions:**

The association between calcification burden and CCTA underestimation is nonlinear and strongly dependent on coronary territory. Since the RCS-derived odds ratio (OR) = 1, crossings depend on the median reference value (CAC = 301) and should be interpreted as markers of risk trajectory rather than absolute diagnostic cutoffs. Integrating total calcium burden with vessel-specific susceptibility may help mitigate the risk of missed severe stenosis on CCTA.

## 1. Introduction

Cardiovascular diseases, particularly coronary artery disease (CAD), remain the leading cause of global morbidity and mortality [1]. Over the past decade, coronary computed tomography angiography (CCTA) has emerged as a first-line non-invasive modality for the evaluation of suspected CAD, primarily due to its exceptional negative predictive value for ruling out anatomically significant stenosis [2,3]. Despite its widespread clinical adoption, the diagnostic accuracy of CCTA is vulnerable to technical and anatomical confounders, including motion artifacts, limited spatial resolution, and heavy calcific plaque burden [4,5].

Coronary artery calcium (CAC), a hallmark of advanced atherosclerosis, presents a persistent challenge in cardiovascular imaging [6,7]. High-density calcified lesions generate partial volume averaging and beam-hardening effects, commonly referred to as blooming artifacts. These artifacts geometrically distort the vessel lumen on CT, resulting in a discrepancy between the non-invasive CCTA assessment and the reference standard, invasive coronary angiography (ICA) [8,9]. While blooming artifacts classically lead to the overestimation of stenosis severity—prompting unnecessary downstream invasive testing—severe calcification can also obscure complex plaque geometries or adjacent non-calcified plaques, paradoxically leading to the underestimation of luminal stenosis [10,11]. Clinically, underestimation represents a critical hazard, as it may result in the inappropriate deferral of revascularization, delayed medical intervention, and a heightened risk of subsequent adverse cardiovascular events [12].

Although the Agatston scoring system is routinely utilized to quantify calcification burden [13], the precise morphological and statistical relationship between CAC score and the risk of CCTA underestimation remains poorly characterized. Previous investigations have predominantly evaluated CCTA accuracy at the patient level, often failing to account for the heterogeneous distribution of calcification across different coronary territories (e.g., the left main versus distal branches) and neglecting the within-patient correlation of multiple vessels [14]. Furthermore, while clinical guidelines caution against interpreting CCTA in the presence of “severe” calcification, they lack empirically derived, robust diagnostic metrics to formally guide when a negative CCTA result should be distrusted [15].

To address these knowledge gaps, this study investigated the nonlinear dose-response association between CAC burden and CCTA underestimation. By employing a comprehensive vessel-level analytical framework incorporating generalized estimating equations (GEE) and restricted cubic splines (RCS) alongside rigorous sensitivity analyses, we sought to elucidate the nonlinear risk trajectories and vessel-specific heterogeneity of calcification artifacts and establish multidimensional, territory-specific risk profiling rather than an isolated diagnostic cutoff to optimize clinical decision-making. We present this article in accordance with the Strengthening the Reporting of Observational Studies in Epidemiology (STROBE) reporting guidelines [16].

## 2. Materials and Methods

### 2.1 Study Population

This retrospective, single-center observational study was conducted at Beijing Friendship Hospital between January 2019 and December 2023. The study population comprised symptomatic and asymptomatic patients who underwent ICA within three months following a CCTA examination. Initial screening included 920 patients with no prior history of confirmed CAD. Following the application of strict inclusion and exclusion criteria, 673 patients (yielding 2692 specific coronary vessel observations) were included in the final analytical cohort (Fig. 1).

**Fig. 1.**
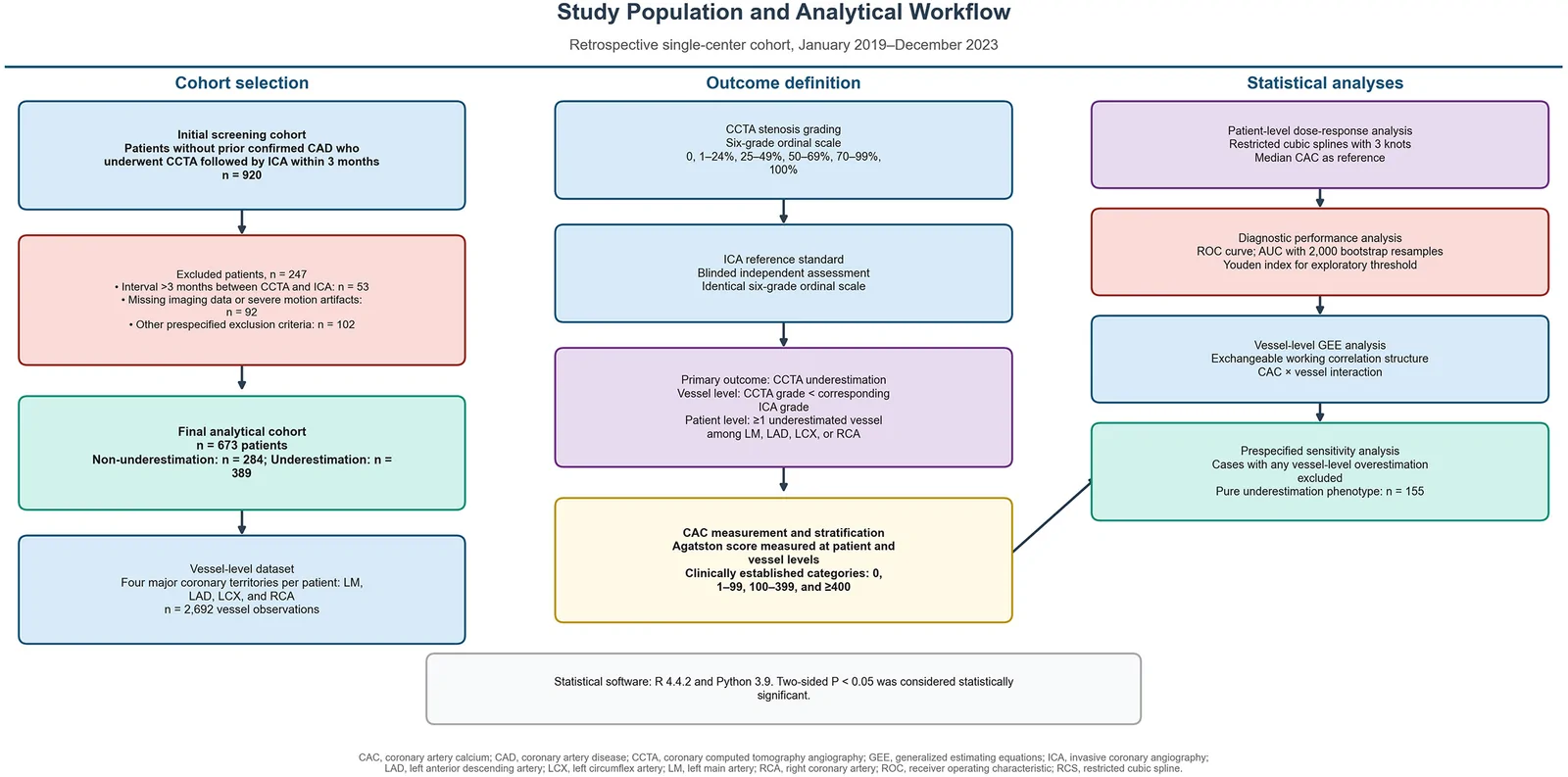
**Flow chart of the study**. CAD, coronary artery disease; CCTA, coronary computed tomography angiography; ICA, invasive coronary angiography; LM, left main; LAD, left anterior descending; LCX, left circumflex; RCA, right coronary artery; CAC, coronary artery calcium.

Inclusion criteria were: (1) age ≥18 years; (2) ICA performed within 90 days of CCTA; (3) no prior formal diagnosis of CAD; and (4) availability of complete demographic, laboratory, and high-quality imaging data. Exclusion criteria included: (1) history of percutaneous coronary intervention (PCI), coronary artery bypass grafting (CABG), or myocardial infarction; (2) documented allergy to iodinated contrast media or advanced renal insufficiency (estimated glomerular filtration rate <30 mL/min/1.73 m^2^); (3) severe symptomatic heart failure (New York Heart Association class III/IV); (4) significant arrhythmias (e.g., atrial fibrillation) precluding adequate electrocardiogram (ECG) gating; (5) active malignancy or pregnancy; and (6) extreme obesity (body mass index [BMI] >40 kg/m^2^).

### 2.2 Coronary Computed Tomography Angiography

CCTA examinations were performed using the latest-generation volumetric CT scanners (Revolution CT, GE Healthcare, Milwaukee, WI, USA). Intravenous iodine-based contrast agents (Iopromide 370 mg I/mL, Bayer Healthcare, Berlin, Germany) were administered via an antecubital vein at a flow rate of 4.5–5.5 mL/s, followed by a 40-mL saline chaser. Scans were acquired using prospective ECG-triggered protocols during a single inspiratory breath-hold to minimize motion artifacts. Standard image reconstruction was performed iteratively (Adaptive Iterative Dose Reduction 3D [AIDR 3D] or Adaptive Statistical Iterative Reconstruction [ASiR-V]) with a 0.5-mm slice thickness. Stenosis severity was evaluated by experienced cardiovascular radiologists based on the maximal luminal diameter reduction. Coronary lesions were categorized into a standardized six-grade ordinal scale: 0 (0%, no stenosis), 1 (1–24%, minimal), 2 (25–49%, mild), 3 (50–69%, moderate), 4 (70–99%, severe), and 5 (100%, total occlusion).

### 2.3 Invasive Coronary Angiography

ICA was performed by experienced interventional cardiologists via radial or femoral artery access using standard catheterization techniques. Intracoronary nitroglycerin (100–200 μg) was routinely administered prior to image acquisition to mitigate vasospasm. Multiple standard angiographic projections (at least two orthogonal views per main vessel) were obtained for optimal visualization of the coronary tree. ICA stenosis grading was assessed quantitatively by two independent specialized readers who were fully blinded to the CCTA findings. Discrepancies were resolved by a third senior interventionalist. Stenosis severity was classified using the identical six-grade system employed for CCTA. For the purposes of this study, ICA served as the definitive anatomical reference standard.

### 2.4 Definition of CCTA Underestimation

To capture anatomical specificity, the primary outcome was defined at both the vessel level and the patient level: (1) Vessel-level underestimation was strictly defined as any instance where the discrete stenosis grade assigned by CCTA was numerically lower than the corresponding ICA grade for the same specific vessel (left main [LM], left anterior descending [LAD], left circumflex [LCX], or right coronary artery [RCA]). (2) Patient-level underestimation was defined as the presence of vessel-level underestimation in at least one of the four major epicardial arteries. Cases where the CCTA grade was equal to or greater than the ICA grade were classified as non-underestimation (encompassing both accurate diagnostic concordance and overestimation). In prespecified sensitivity analyses, patients exhibiting overestimation in any of the four major vessels (n = 518) were completely excluded to isolate the pure underestimation phenotype in the restricted cohort (n = 155).

### 2.5 Measurement of Coronary Artery Calcification

Non-contrast prospectively ECG-gated scanning was performed prior to CCTA for calcium scoring (tube voltage 120 kV, slice thickness 3.0 mm). CAC scores were quantified utilizing the standard Agatston methodology [13], wherein calcified lesions were identified by a density threshold of ≥130 Hounsfield Units (HU) spanning an area of at least 1 mm^2^ (three contiguous pixels). The patient-level total CAC score was calculated as the sum of all individual vessel scores. For analytical purposes, both patient-level and vessel-specific CAC scores were stratified into four clinically established categories: 0, 1–99, 100–399, and ≥400.

### 2.6 Statistical Analysis

Baseline characteristics were compared between patients with and without underestimation using the Mann-Whitney U test for continuous variables (presented as median and interquartile range [IQR]) and the Chi-square or Fisher’s exact test for categorical variables (presented as frequencies and percentages). Missing data were minimal (<1%) and handled using complete case analysis.

Patient-Level Dose-Response Analysis: The continuous nonlinear association between total CAC score and patient-level underestimation was evaluated using Harrell RCS with three knots located at the 10th, 50th, and 90th percentiles of the CAC distribution. The median CAC score served as the reference point. RCS analyses were used solely to describe the continuous risk trajectory; no RCS-derived odds ratio (OR) = 1 crossing points were utilized as diagnostic thresholds. Three nested logistic regression models were constructed: Model 1 (unadjusted), Model 2 (adjusted for age and sex), and Model 3 (fully adjusted for age, sex, BMI, hypertension, diabetes, hyperlipidemia, smoking, alcohol consumption, history of angina, heart rate, systolic blood pressure [SBP], diastolic blood pressure [DBP], triglycerides [TG], creatinine [Cr], blood urea nitrogen [BUN], total cholesterol [TC], high-sensitivity C-reactive protein [hs-CRP], low-density lipoprotein cholesterol [LDL-C], high-density lipoprotein cholesterol [HDL-C], alanine aminotransferase [ALT], aspartate aminotransferase [AST], and myocardial bridge). Likelihood-ratio tests were employed to ascertain both the overall statistical significance and the presence of nonlinearity.

Sensitivity Analysis: To explicitly account for the confounding effect of classical blooming artifacts, a prespecified sensitivity analysis was conducted by completely excluding patients who exhibited overestimation in any vessel territory. Within this restricted cohort, the continuous nonlinear association between total CAC score and the isolated risk of underestimation was re-evaluated using the aforementioned RCS framework.

Vessel-Level GEE Analysis: To robustly account for the intrinsic within-patient correlation among the four coronary territories, vessel-level underestimation was analyzed using GEE with a logit link function, an exchangeable working correlation matrix, and robust Huber-White sandwich standard error estimators. GEE models incorporated patient-level CAC, vessel-specific CAC, and specific vessel territory (LM, LAD, LCX, RCA) as primary predictors. Interaction terms (CAC × Vessel) were introduced to formally test whether the magnitude of calcification-induced artifact differed across anatomical locations.

All statistical analyses were executed using R software (version 4.4.2, R Foundation for Statistical Computing, Vienna, Austria) equipped with the rms, geepack, and pROC packages, as well as Python (version 3.9, Python Software Foundation, Wilmington, DE, USA) utilizing the NumPy, SciPy, pandas, statsmodels, and scikit-learn libraries. A two-sided *p*-value < 0.05 was considered statistically significant.

## 3. Results

### 3.1 Illustrative Cases

To enhance clinical interpretability, representative cases demonstrating diagnostic discordance are presented in Fig. 2. Fig. 2A illustrates a case of severe underestimation in a 72-year-old male with a high total CAC score (581.55). Due to severe calcified blooming and partial volume averaging, CCTA suggested only moderate luminal stenosis in the proximal-to-mid LAD; however, subsequent ICA revealed diffuse severe luminal stenosis in the corresponding segments. Fig. 2B demonstrates a contrasting scenario in a 49-year-old male (CAC score 321.47), where CCTA assessed mild luminal stenosis in the LAD and LCX, but ICA identified moderate stenosis. These cases intuitively highlight how calcification geometry can obscure critical luminal narrowing, leading readers to underestimate the true anatomical severity.

**Fig. 2.**
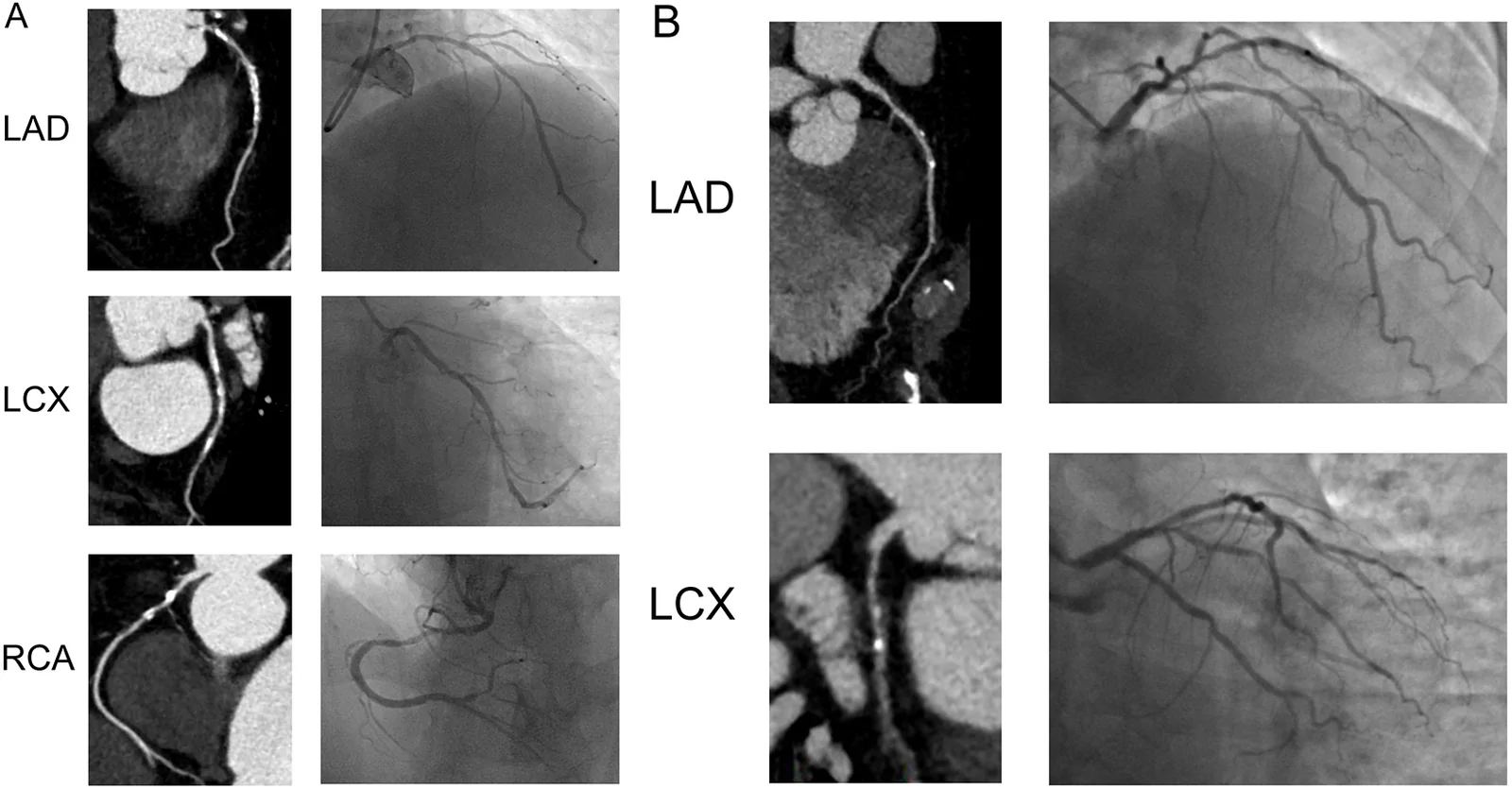
**Representative cases demonstrating diagnostic discordance**. (A) 72-year-old male with a calcification score of 581.55. CCTA identified moderate (grade 3) luminal stenosis in the proximal and mid-segments of the left anterior descending artery. However, ICA revealed diffuse severe (grade 4) luminal stenosis in the same segments. Moderate luminal stenoses were identified in the left circumflex artery and right coronary artery by both CCTA and ICA. (B) 49-year-old male with a calcification score of 321.47. CCTA identified mild (grade 2) luminal stenosis in the left anterior descending artery and left circumflex artery. However, ICA revealed moderate (grade 3) luminal stenosis in the left anterior descending artery and left circumflex artery.

### 3.2 Baseline Characteristics

A total of 673 patients who underwent both CCTA and ICA were included in the final analysis. Based on the vessel-level definition, any-vessel CCTA underestimation occurred in 389 patients (57.8%). The baseline clinical and demographic characteristics stratified by the presence of patient-level CCTA underestimation are summarized in Table 1. Patients with underestimation had significantly higher triglyceride levels compared to those without underestimation (1.36 vs. 1.19 mmol/L, *p* < 0.001). The underestimation group exhibited a significantly higher calcification burden, as evidenced by a higher median total CAC score (316.3 vs. 270.0, *p* = 0.025) and a distinct distribution across clinical CAC categories (*p* = 0.003). There were no significant differences between the two groups regarding age, sex, BMI, blood pressure, or the prevalence of major comorbidities such as hypertension, diabetes, and history of angina.

**Table 1.** **Baseline characteristics according to patient-level CCTA underestimation**.

Variable	Non-underestimation (n = 284)	Underestimation (n = 389)	*p* value
Age (years)	64 (58, 70)	65 (59, 71)	0.382
BMI (kg/m^2^)	25.4 (23.2, 28.0)	25.9 (23.6, 28.1)	0.262
Heart rate (bpm)	70 (63, 77.2)	71 (65, 78)	0.056
SBP (mmHg)	129.5 (119, 139)	132 (121, 143)	0.078
DBP (mmHg)	74 (66.8, 82)	75 (68, 84)	0.144
TG (mmol/L)	1.19 (0.91, 1.63)	1.36 (0.97, 1.94)	<0.001
Creatinine (μmol/L)	71.0 (60.6, 81.8)	72.4 (63, 82.7)	0.373
BUN (mmol/L)	5.29 (4.63, 6.21)	5.35 (4.43, 6.20)	0.850
TC (mmol/L)	3.87 (3.34, 4.58)	4.01 (3.33, 4.73)	0.236
Hs-CRP (mg/L)	0.98 (0.41, 2.66)	1.03 (0.44, 2.97)	0.726
LDL-C (mmol/L)	2.23 (1.85, 2.84)	2.40 (1.89, 2.89)	0.091
HDL-C (mmol/L)	1.06 (0.90, 1.22)	1.02 (0.87, 1.20)	0.057
ALT (U/L)	18 (14, 26)	17 (13, 24)	0.144
AST (U/L)	19.6 (16.3, 23.9)	19 (16.4, 23.4)	0.379
Total CAC score	270.0 (31.0, 794.0)	316.3 (104.7, 829.5)	0.025
Male (n, %)	181 (63.7)	267 (68.6)	0.183
Hypertension (n, %)	207 (72.9)	292 (75.1)	0.524
Diabetes (n, %)	98 (34.5)	160 (41.1)	0.081
Hyperlipidemia (n, %)	159 (56.0)	209 (53.7)	0.561
Smoking (n, %)	153 (53.9)	210 (54.0)	0.977
Alcohol (n, %)	118 (41.5)	170 (43.7)	0.577
History of angina (n, %)	216 (76.1)	287 (73.8)	0.502
Myocardial bridge (n, %)	51 (18.0)	52 (13.4)	0.102
CAC 0 (n, %)	35 (12.3)	21 (5.4)	
CAC 1–99 (n, %)	65 (22.9)	75 (19.3)	
CAC 100–399 (n, %)	66 (23.2)	119 (30.6)	
CAC ≥400 (n, %)	118 (41.5)	174 (44.7)	
CAC_4 level overall			0.003

Notes: ALT, alanine aminotransferase; AST, aspartate aminotransferase; BMI, body mass index; BUN, blood urea nitrogen; DBP, diastolic blood pressure; HDL-C, high-density lipoprotein cholesterol; hs-CRP, high-sensitivity C-reactive protein; IQR, interquartile range; LDL-C, low-density lipoprotein cholesterol; SBP, systolic blood pressure; TC, total cholesterol; TG, triglycerides.

### 3.3 Underestimation Rates Across CAC Categories and Coronary Vessels

The prevalence of CCTA underestimation demonstrated a nonlinear trajectory with increasing CAC burden and exhibited marked heterogeneity across different coronary territories (Table 2). At the patient level, the rate of any-vessel underestimation increased from 37.5% in patients with zero CAC to 64.3% in those with a CAC score of 100–399, before slightly plateauing at 59.6% in patients with severe calcification (CAC ≥400) (*p* for trend = 0.007). Regarding vessel-specific susceptibility, the LM artery was rarely underestimated, with an overall occurrence rate of only 3.0%. In stark contrast, underestimation was highly prevalent in the LAD (27.9%), LCX (29.3%), and RCA (22.4%).

**Table 2.** **Patient-level CAC categories and vessel-specific underestimation rates**.

CAC Category	N	Underestimation Vessel				
		Any vessel	LM	LAD	LCX	RCA
0	56	21 (37.5%)	1 (1.8%)	12 (21.4%)	10 (17.9%)	7 (12.5%)
1–99	140	75 (53.6%)	0 (0.0%)	45 (32.1%)	35 (25.0%)	32 (22.9%)
100–399	185	119 (64.3%)	4 (2.2%)	48 (25.9%)	54 (29.2%)	55 (29.7%)
≥400	292	174 (59.6%)	15 (5.1%)	83 (28.4%)	98 (33.6%)	57 (19.5%)
Total	673	389 (57.8%)	20 (3.0%)	188 (27.9%)	197 (29.3%)	151 (22.4%)
*p* for trend		0.007	0.007	0.805	0.007	0.849

Notes: Underestimation was defined as a lower stenosis grade on CCTA compared to ICA. “Any vessel” indicates the presence of underestimation in at least one of the four major epicardial coronary arteries. The *p* for trend was calculated using the Cochran-Armitage test for trend.

### 3.4 Impact of Patient-Level vs. Vessel-Level Calcification

To delineate the anatomical specificities, we evaluated the underestimation rates stratified by both patient-level total CAC and vessel-specific local CAC (Table 3). The influence of calcification varied substantially among the vessels. For the LCX, the underestimation rate significantly escalated with increasing local vessel CAC burden (*p* for trend = 0.002), rising from 19.4% in segments with zero local calcium to 37.2% and 32.5% in mild and moderate local calcification, respectively. Conversely, for the LAD and RCA, the local CAC categories did not show a monotonic trend with underestimation rates, implying that factors beyond isolated local calcification—such as total calcification-induced generalized artifact or vessel-specific anatomical geometry—may contribute to the discrepancies between CCTA and ICA.

**Table 3.** **Vessel-specific CCTA underestimation rates stratified by patient-level and vessel-level CAC categories**.

Vessel	CAC Category	By patients (n, %)		By vessels (n, %)	
		N	Underestimation	N	Underestimation
LM	All	673	20 (3.0%)	673	20 (3.0%)
	0	56	1 (1.8%)	356	5 (1.4%)
	1–99	140	0 (0.0%)	212	8 (3.8%)
	100–399	185	4 (2.2%)	93	5 (5.4%)
	≥400	292	15 (5.1%)	12	2 (16.7%)
	*p* for trend		0.007		0.001
LAD	All	673	188 (27.9%)	673	188 (27.9%)
	0	56	12 (21.4%)	85	21 (24.7%)
	1–99	140	45 (32.1%)	186	58 (31.2%)
	100–399	185	48 (25.9%)	265	78 (29.4%)
	≥400	292	83 (28.4%)	137	31 (22.6%)
	*p* for trend		0.805		0.474
LCX	All	673	197 (29.3%)	673	197 (29.3%)
	0	56	10 (17.9%)	258	50 (19.4%)
	1–99	140	35 (25.0%)	247	92 (37.2%)
	100–399	185	54 (29.2%)	126	41 (32.5%)
	≥400	292	98 (33.6%)	42	14 (33.3%)
	*p* for trend		0.007		0.002
RCA	All	673	151 (22.4%)	673	151 (22.4%)
	0	56	7 (12.5%)	185	34 (18.4%)
	1–99	140	32 (22.9%)	221	69 (31.2%)
	100–399	185	55 (29.7%)	153	33 (21.6%)
	≥400	292	57 (19.5%)	114	15 (13.2%)
	*p* for trend		0.849		0.186
All vessels	All	2692	556 (20.7%)	2692	556 (20.7%)

Notes: This table compares the occurrence of stenosis underestimation in specific coronary arteries grouped by the patient’s total CAC score (By patients) versus the localized CAC score within that specific vessel (By vessels). The *p* for trend evaluates the monotonic change across the four CAC categories.

### 3.5 Nonlinear Association Between Total CAC and Underestimation Risk

RCS analysis revealed a significant, nonlinear dose-response relationship between the continuous total CAC score and the risk of patient-level CCTA underestimation (Fig. 3). Across the three nested models, the overall associations remained statistically significant: Model 1, unadjusted (*p*-overall = 0.012); Model 2, adjusted for age and sex (*p*-overall = 0.021); and Model 3, fully adjusted (*p*-overall = 0.014). In the fully adjusted model (Model 3), there was strong evidence of nonlinearity (*p*-nonlinear = 0.004). Using the median CAC score of 301 as the reference value, the RCS curves crossed the reference line of OR = 1 at two CAC values in each model, reflecting the nonlinear configuration of the association.

**Fig. 3.**
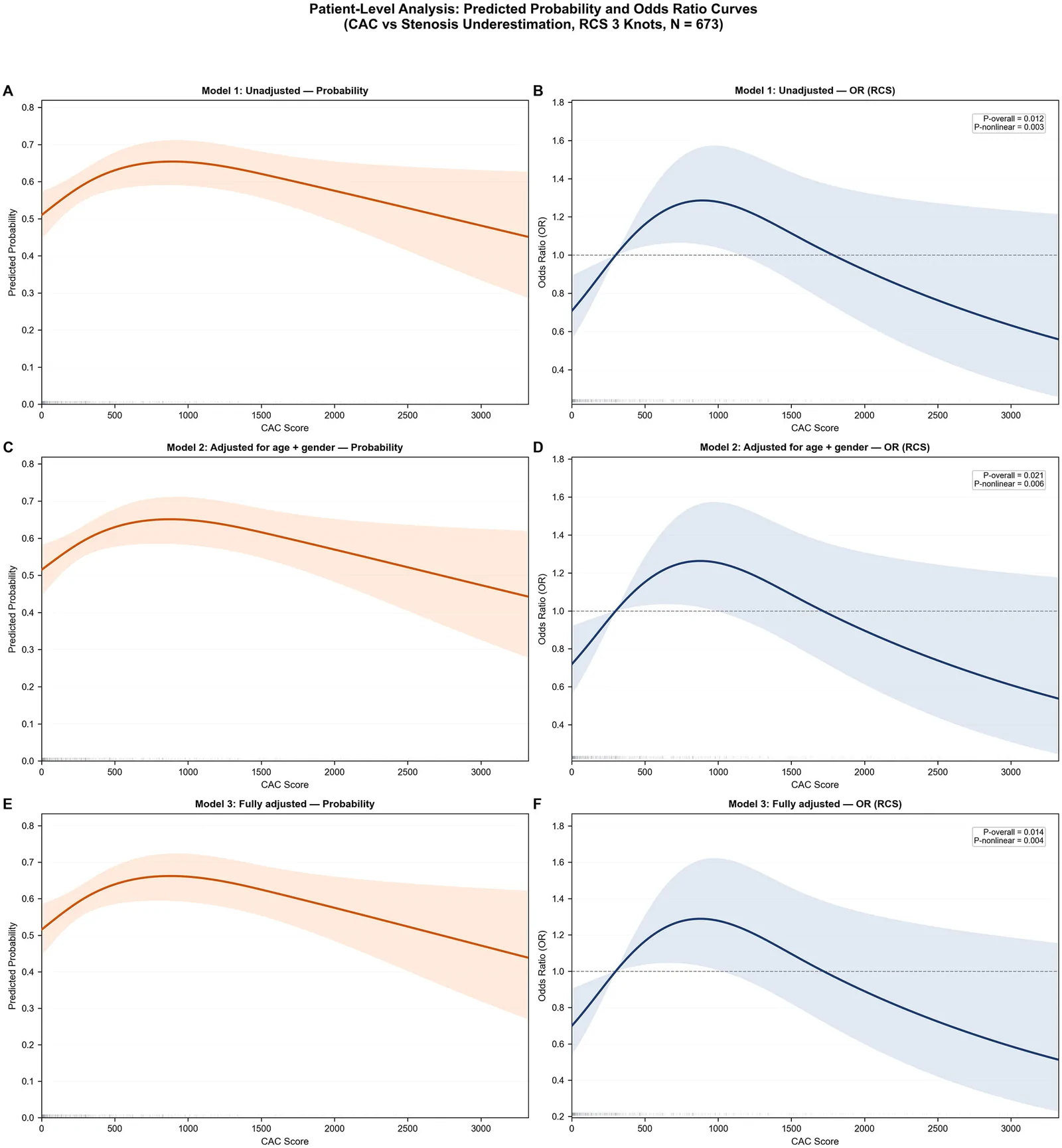
**Adjusted restricted cubic spline curve for total CAC and patient-level CCTA underestimation**. (A) Predicted probability of CCTA underestimation in Model 1 (unadjusted). (B) Corresponding OR curve for Model 1. (C) Predicted probability of CCTA underestimation in Model 2, adjusted for age and sex. (D) Corresponding OR curve for Model 2. (E) Predicted probability of CCTA underestimation in Model 3, fully adjusted for demographic and clinical covariates. (F) Corresponding OR curve for Model 3. RCS analysis was used to visualize the nonlinear dose-response relationship between the continuous patient-level CAC score and the risk of CCTA underestimation. In (B,D,F), the solid line represents the estimated OR and the shaded area represents the 95% confidence interval. The median CAC score (301) serves as the reference point (OR = 1.00). *p*-values for the overall association (*p*-overall) and nonlinearity (*p*-nonlinear) are provided.

Importantly, these OR = 1 crossing points indicate only where the estimated risk equals the reference risk at CAC = 301 and were not interpreted as diagnostic thresholds or clinical cutoffs. They were not used for receiver operating characteristic (ROC) analysis or any diagnostic performance evaluation. The risk of underestimation began to increase substantially as the total CAC score exceeded the median reference value of 301. For instance, the adjusted OR reached 1.09 (95% CI: 1.02–1.16) at a CAC score of 400 and further escalated to 1.28 (95% CI: 1.04–1.59) at a CAC score of 800.

### 3.6 Vessel-Level Nonlinear Association Between Local CAC and Underestimation Risk

Vessel-level restricted RCS analyses further demonstrated substantial heterogeneity in the association between local vessel CAC burden and CCTA underestimation across coronary territories (Fig. 4). In the LM artery, local CAC was significantly associated with underestimation across all three models, with no evidence of nonlinearity (Model 1: *p*-overall = 0.028, *p*-nonlinear = 1.000; Model 2: *p*-overall = 0.050, *p*-nonlinear = 1.000; Model 3: *p*-overall = 0.029, *p*-nonlinear = 1.000). For the RCA, local CAC also showed a significant overall association with underestimation in all models, while the relationship remained largely linear (Model 1: *p*-overall = 0.015, *p*-nonlinear = 0.750; Model 2: *p*-overall = 0.018, *p*-nonlinear = 0.778; Model 3: *p*-overall = 0.020, *p*-nonlinear = 0.945). In contrast, the LAD and LCX did not show statistically significant overall or nonlinear associations after adjustment (LAD Model 3: *p*-overall = 0.237, *p*-nonlinear = 0.226; LCX Model 3: *p*-overall = 0.332, *p*-nonlinear = 0.221). These findings suggest that the effect of local calcification on stenosis underestimation is not uniform across coronary arteries and may be more evident in specific vascular territories such as the LM and RCA.

**Fig. 4.**
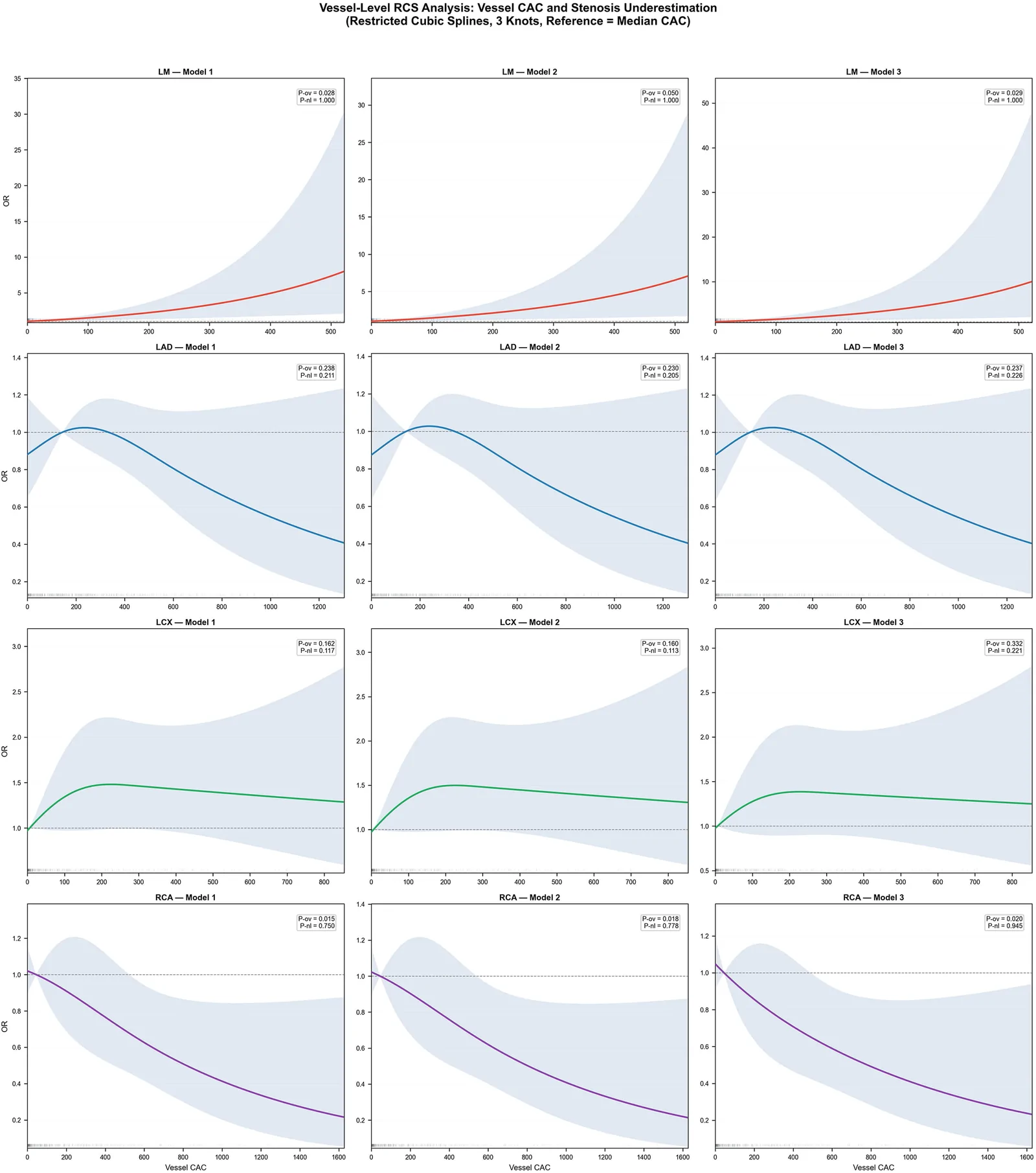
**Vessel-specific restricted cubic spline curves for the association between local vessel CAC score and CCTA stenosis underestimation**. Vessel-level RCS curves demonstrate the association between local vessel CAC score and the risk of CCTA stenosis underestimation in the LM, LAD, LCX, and RCA. Three models are shown for each vessel: Model 1, unadjusted; Model 2, adjusted for age and sex; and Model 3, fully adjusted for demographic and clinical covariates. The median vessel-specific CAC score was used as the reference value. The solid line represents the estimated OR, and the shaded area represents the 95% confidence interval. *p*-overall evaluates the overall association, whereas *p*-nonlinear evaluates departure from linearity.

### 3.7 Vessel-Specific Heterogeneity and GEE Analysis

The susceptibility to CCTA underestimation was highly variable across different coronary territories (Fig. 5). GEE models, which robustly accounted for the within-patient clustering of the four major vessels, confirmed that both patient-level CAC burden and the specific vessel territory were independent predictors of local underestimation (Fig. 6). In the fully adjusted model, compared to patients with zero CAC, those with a total CAC of 1–99, 100–399, and ≥400 had significantly elevated odds of vessel-level underestimation (OR = 1.75, *p* = 0.039; OR = 1.98, *p* = 0.008; and OR = 2.01, *p* = 0.008, respectively). Furthermore, compared to the LM artery, the LAD, LCX, and RCA exhibited profoundly higher underestimation risks (all *p* < 0.001). A highly significant interaction between patient-level CAC and vessel type was observed (*p* < 0.001), indicating that the detrimental impact of calcification on CCTA accuracy is not uniform but heavily dependent on the specific coronary anatomy. Complementary GEE analyses that instead used vessel-level (local) CAC as the primary exposure produced a consistent pattern. In the unadjusted vessel-CAC model, vessels with a local CAC of 1–99 and 100–399 had significantly elevated odds of underestimation compared with a vessel-CAC of 0 (OR = 2.04, 95% CI: 1.56–2.66, *p* < 0.001; OR = 1.57, 95% CI: 1.15–2.13, *p* = 0.004, respectively), whereas a vessel-CAC ≥400 was not independently predictive (OR = 1.06, 95% CI: 0.71–1.57, *p* = 0.79). After full adjustment for demographic and clinical covariates, the estimates for the two lower vessel-CAC strata remained essentially unchanged (OR = 1.99, 95% CI: 1.51–2.60 for 1–99; OR = 1.54, 95% CI: 1.11–2.13 for 100–399), and the vessel-territory effects versus the LM artery remained profoundly elevated (LAD OR = 12.26, LCX OR = 13.44, RCA OR = 9.32; all *p* < 0.001). A significant vessel-CAC × vessel-territory interaction was also detected (*p* = 0.025), further indicating that the impact of local calcification on CCTA accuracy is territory-dependent rather than a uniform, dose-linear effect.

**Fig. 5.**
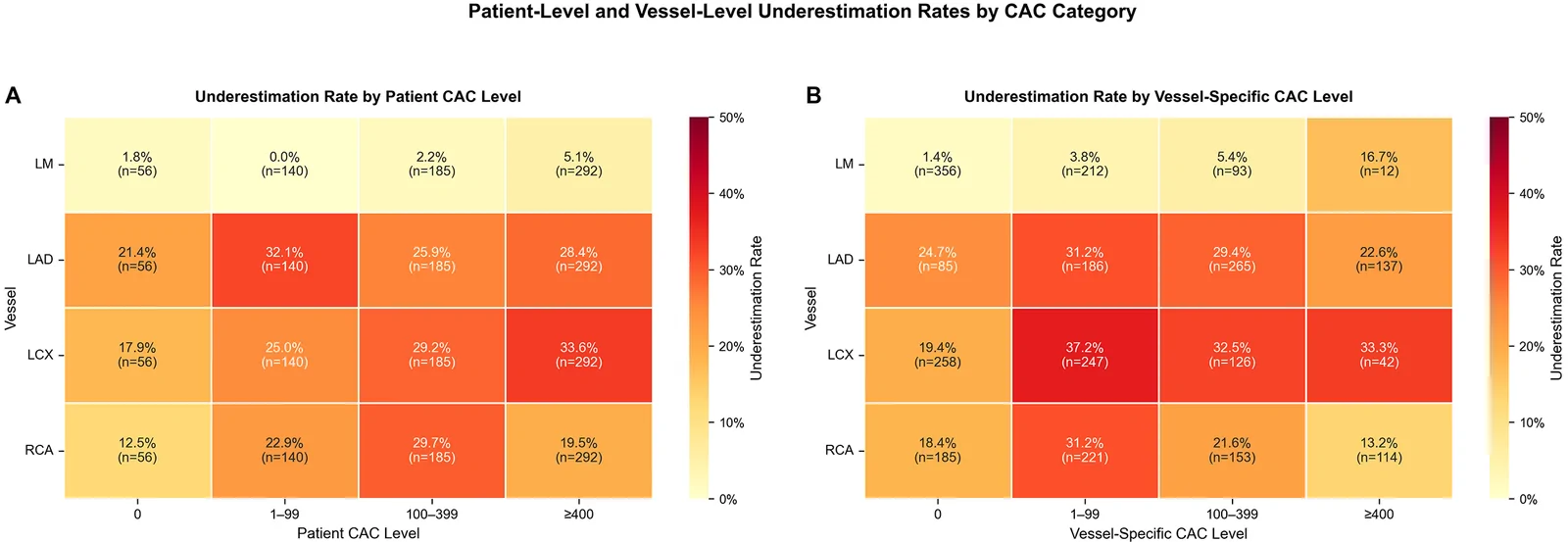
**Patient- and vessel-specific underestimation heatmap by CAC category**. (A) CCTA underestimation rates for each coronary vessel stratified by patient-level total CAC category. (B) CCTA underestimation rates for each coronary vessel stratified by vessel-specific CAC category. The color intensity corresponds to the underestimation percentage, providing a visual representation of susceptibility to underestimation across different levels of calcification burden.

**Fig. 6.**
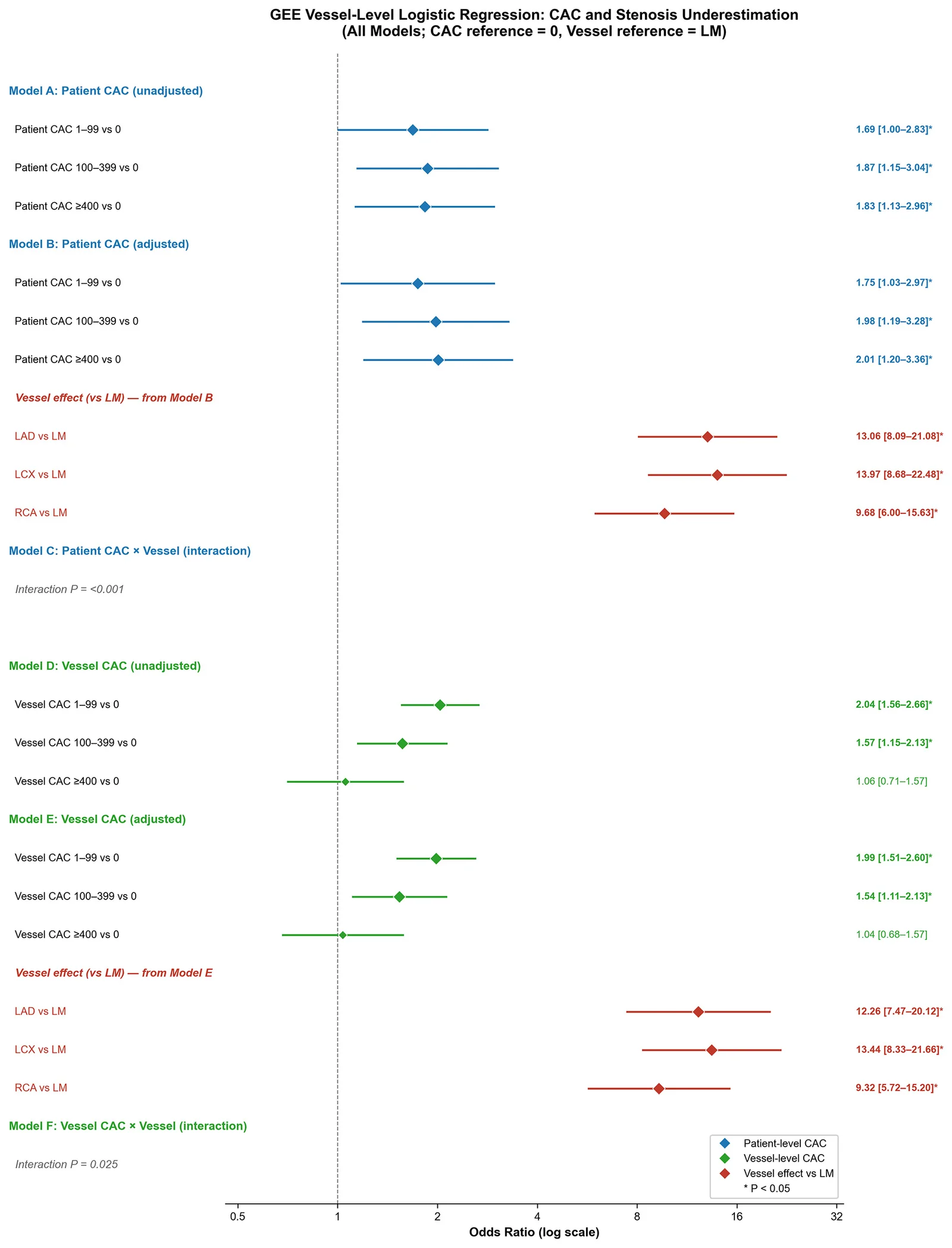
**Forest plot of vessel-level GEE results**. The forest plot presents ORs and 95% confidence intervals for vessel-level CCTA underestimation derived from six GEE models with an exchangeable working correlation. The models account for the within-patient clustering of four coronary territories (LM, LAD, LCX, RCA). Model A: patient-level CAC (unadjusted); Model B: patient-level CAC, fully adjusted for age, sex, BMI, hypertension, diabetes, hyperlipidemia, smoking, alcohol, angina history, heart rate, SBP, DBP, TG, Cr, BUN, TC, hs-CRP, LDL-C, HDL-C, ALT, AST, and myocardial bridge; Model C: patient-CAC × vessel-territory interaction (Wald P shown). Model D: vessel-level (local) CAC (unadjusted); Model E: vessel-level CAC, fully adjusted for the same covariates as Model B; Model F: vessel-CAC × vessel-territory interaction. CAC = 0 is the reference for both patient-level and vessel-level CAC categories, and the LM artery is the reference for the vessel-territory effect (highlighted in red). Diamonds denote OR point estimates and horizontal bars denote the 95% CI; asterisks (*) indicate *p* < 0.05.

### 3.8 Subgroup Analysis

Subgroup analyses were conducted to evaluate the consistency of the association across diverse clinical profiles (Fig. 7). The association between CAC burden and CCTA underestimation remained largely consistent across most demographic and clinical strata, including age, sex, BMI, and diabetes status. However, TG emerged as a significant effect modifier (*p*-interaction = 0.032). The association was particularly robust in patients with normal TG levels (<1.7 mmol/L; OR = 2.65, 95% CI: 1.63–4.31), whereas it was attenuated and non-significant in those with elevated TG levels (≥1.7 mmol/L; OR = 0.96, 95% CI: 0.45–2.07).

**Fig. 7.**
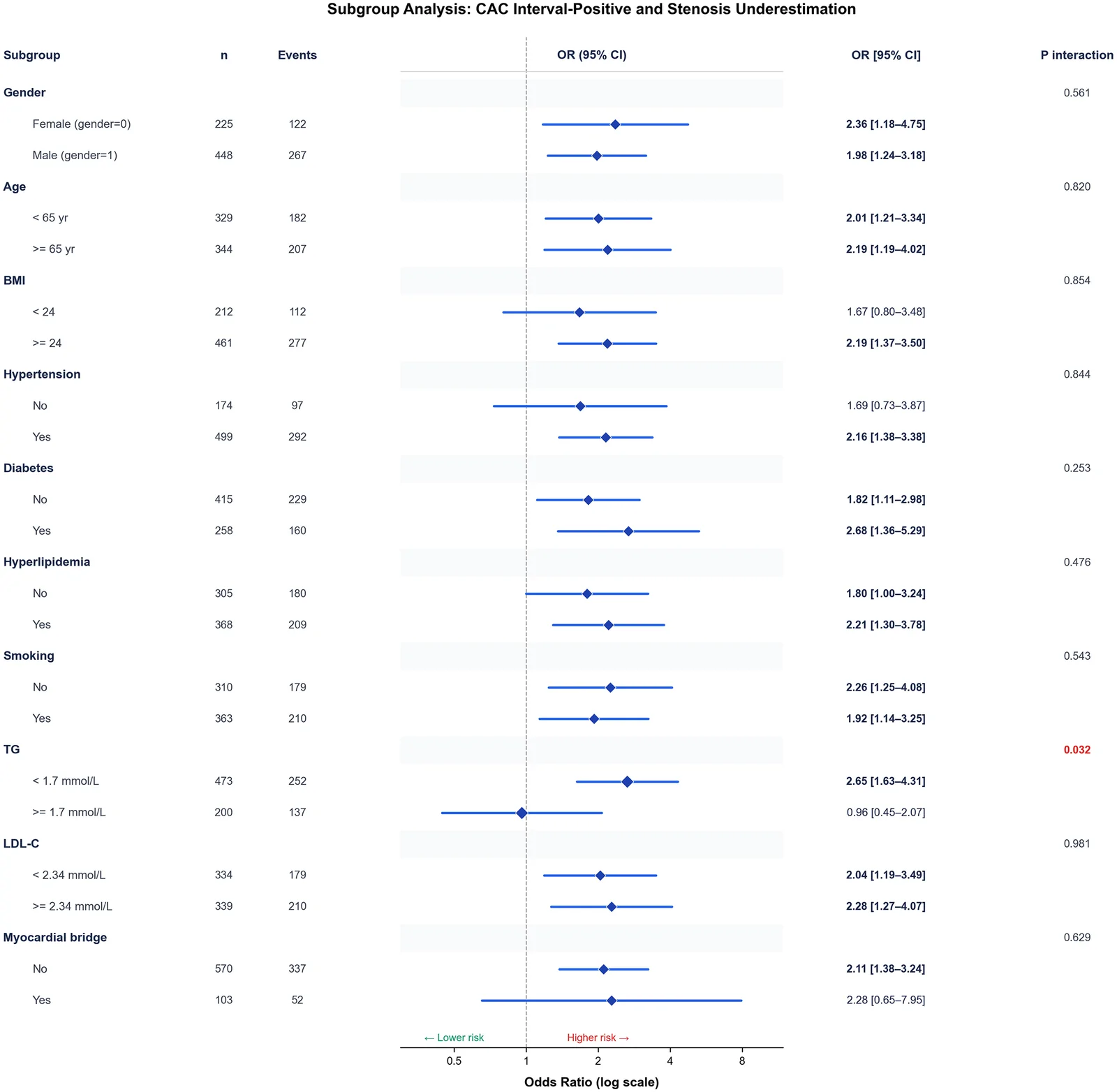
**Subgroup forest analysis for the association between CAC and CCTA underestimation**. This forest plot displays the ORs and 95% confidence intervals for the association between a positive CAC interval rule and patient-level CCTA underestimation across various pre-specified clinical subgroups. Multivariable logistic regression (adjusted for Model 3 covariates) was used within each stratum. The *p*-value for interaction (*p*-interaction) indicates whether the effect of CAC on underestimation differs significantly between the strata of each subgroup.

### 3.9 Sensitivity Analysis

To validate the robustness and clinical interpretability of our findings, we performed a prespecified sensitivity analysis by excluding all patients with any CCTA overestimation. Patient-level overestimation was defined as the presence of overestimation in at least one of the four major coronary vessels, including the LM, LAD, LCX, and RCA. After excluding 518 patients with any-vessel overestimation, the sensitivity cohort included 155 patients without any overestimation, among whom 113 had CCTA underestimation, corresponding to an event rate of 72.9%.

In this restricted cohort, RCS analysis demonstrated a significant nonlinear association between total CAC score and the predicted probability of CCTA underestimation (*p*-overall = 0.012; *p*-nonlinear = 0.005) (Fig. 8). As shown in Fig. 8A, the predicted probability of underestimation did not increase in a simple linear manner across the CAC spectrum, but instead varied dynamically with increasing CAC burden. The corresponding OR curve in Fig. 8B showed that the estimated odds crossed the reference line of OR = 1 at a CAC score of approximately 300. This value indicates the point at which the estimated risk equals the reference risk in the spline model. Importantly, this OR = 1 crossing point was treated only as a graphical reference for interpreting the nonlinear risk trajectory and was not used as a diagnostic threshold, clinical cutoff, or basis for any ROC-derived performance evaluation (**Supplementary Figs**.** 1**,**2**).

**Fig. 8.**
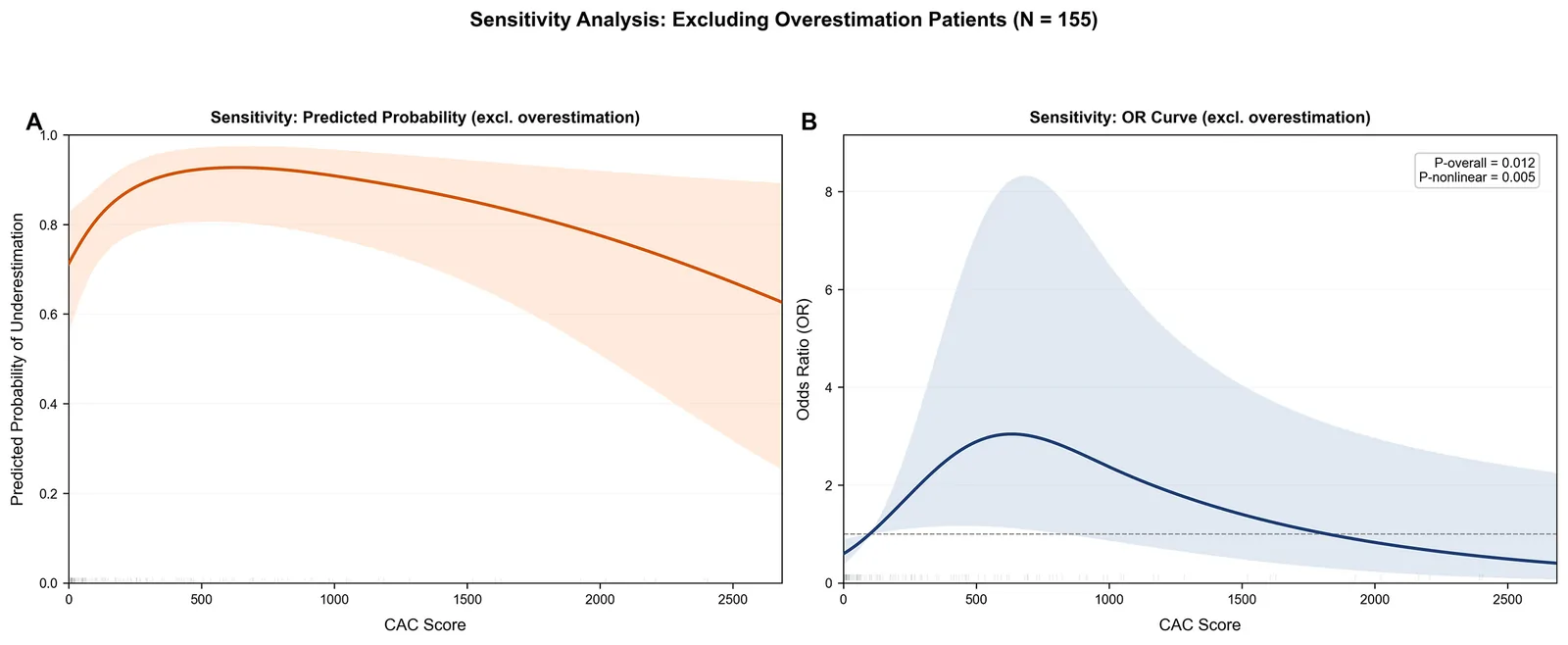
**Sensitivity analysis of the nonlinear association between CAC and CCTA underestimation after excluding patients with overestimation**. This figure presents the RCS analysis after restricting the cohort to patients without any CCTA overestimation (N = 155). (A) shows the predicted probability of CCTA underestimation across the continuous total CAC score, and (B) shows the corresponding OR curve. The analysis demonstrated a significant overall association and nonlinearity between total CAC score and underestimation risk (*p*-overall = 0.012; *p*-nonlinear = 0.005). The OR = 1 crossing point at approximately 300 indicates where the estimated risk equals the spline reference risk and is provided only as a graphical reference. It should not be interpreted as a validated diagnostic cutoff or clinical threshold.

Taken together, these sensitivity findings suggest that concurrent overestimation may obscure the association between CAC burden and pure underestimation risk in the full cohort. After removing patients with any-vessel overestimation, the relationship between CAC and underestimation became more apparent, while remaining clearly nonlinear. These results further support our interpretation that CAC is best regarded as a risk-stratification marker to be interpreted together with vessel-specific anatomy and clinical context, rather than as an isolated threshold-based diagnostic tool.

## 4. Discussion

This study demonstrates that the relationship between CAC burden and the underestimation of stenosis severity on CCTA is complex, highly nonlinear, and profoundly dependent on the specific vascular territory. While elevated CAC is conventionally recognized as a primary driver of CCTA overestimation due to blooming artifacts, our vessel-level GEE analysis reveals that severe calcification simultaneously elevates the risk of clinically critical underestimation. The RCS analysis confirmed a highly significant nonlinear dose-response relationship, indicating that the probability of underestimation accelerates dynamically once the total CAC score exceeds the population median. Given this inherent non-linearity and the profound anatomical heterogeneity confirmed by our GEE models, attempting to define a single diagnostic threshold for the total CAC score is clinically inadequate. Instead, vessel-specific risk profiling provides a much more accurate representation of diagnostic discordance. Collectively, these findings indicate that while a single CAC threshold cannot strictly diagnose diagnostic discordance, severe calcification serves as a critical systemic risk modifier that warrants heightened clinical suspicion for missed severe stenosis, particularly in specific coronary anatomies.

The paradox of calcification-induced underestimation requires careful mechanistic interpretation. Heavy calcification generates beam-hardening and blooming artifacts, which typically enlarge the apparent volume of the plaque and obscure the residual lumen, causing readers to overcall stenosis [7,10]. Consequently, a previous study has predominantly focused on the reduced specificity and positive predictive value of CCTA in highly calcified vessels [17]. However, the sensitivity analysis in our study provides a distinct mechanistic insight: when patients with any-vessel overestimation were excluded, the pure underestimation phenotype revealed a highly significant, unobstructed nonlinear probability curve across the CAC spectrum. This confirms that the traditional blooming artifact noise (overestimation) fundamentally masks a concurrent, distinct diagnostic failure phenotype (underestimation). In vessels with massive, circumferential, or eccentric calcification, the true lumen may be completely indistinguishable. In such scenarios, clinical readers or automated interpretation software, conditioned to mentally “subtract” the blooming effect, may overcompensate. If distal contrast enhancement is preserved, they may erroneously infer luminal patency, systematically under-grading the lesion [18,19].

The complex nonlinear dynamics observed in our cohort also highlight the challenge of defining an absolute diagnostic threshold for CAC. While the CAC values corresponding to an odds ratio of 1.0 (OR = 1) on the RCS curves provide graphical reference points that help visualize where risk dynamics shift, we emphasize that these intersections should not be interpreted as validated diagnostic cutoffs. Establishing definitive, universally applicable continuous CAC thresholds for CCTA interpretation remains fundamentally flawed due to the interplay of non-linearity and anatomical heterogeneity. Instead of relying on a single continuous cutoff, alternative structural approaches may prove more clinically viable. For instance, the traditional four-tier ordinal CAC classification system, or the integration of vessel-specific risk adjustments—as demonstrated by our GEE models—can provide a more reliable and multidimensional framework for risk stratification. These methods accommodate the anatomic and mechanistic heterogeneity of calcified plaques better than an isolated mathematical threshold.

Anatomical heterogeneity emerged as a dominant determinant of CCTA performance. Generalized estimating equation models demonstrated a highly significant interaction between calcification and vessel type. The LM artery was nearly immune to underestimation (3.0% occurrence), likely due to its larger luminal caliber, proximal location, and reduced susceptibility to cardiac motion artifacts. In contrast, the LAD, LCX, and RCA exhibited massive increases in underestimation odds. These distal vessels traverse complex, tortuous courses and are subject to continuous, high-velocity dynamic motion during the cardiac cycle. When heavy local calcification is superimposed on these smaller-caliber, high-motion segments, the spatial and temporal resolution limits of standard multidetector CT are frequently exceeded [20,21]. Our vessel-level analysis implies that interpreting radiologists must apply distinct thresholds of confidence depending on the specific coronary branch being evaluated, rather than relying on a generalized whole-heart interpretation.

The interaction between triglyceride levels and calcification burden highlights the complex interplay of mixed plaque morphologies. Subgroup analysis revealed that the association between CAC and underestimation was entirely attenuated in patients with elevated triglycerides (≥1.7 mmol/L). Hypertriglyceridemia is robustly associated with the progression of lipid-rich, non-calcified, and highly vulnerable atherosclerotic plaques [22]. In the presence of extensive non-calcified plaque burden, positive vascular remodeling often occurs, and the low-attenuation lipid core presents distinct imaging challenges on CCTA, frequently predisposing the evaluation to overestimation rather than underestimation [23]. The competing presence of prominent lipid-rich plaques may offset the specific visual phenomena that lead to the underestimation of heavily calcified lesions, explaining the loss of the CAC-underestimation association in this specific metabolic subgroup.

Clinically, our findings strongly advocate for an integrated, territory-specific diagnostic pathway. The historical reliance on a singular absolute threshold (e.g., Agatston >400) to entirely reject CCTA utility is overly simplistic. Instead, when a CCTA scan reveals severe CAC but interprets distal segments (particularly in the LAD or LCX) as merely “mild-to-moderate”, clinicians must integrate the patient’s pre-test clinical probability. Advanced diagnostic adjuncts, such as CCTA-derived fractional flow reserve (FFR-CT) or stress myocardial perfusion imaging, should be aggressively considered to rule out functionally significant but anatomically underestimated lesions [24,25]. Furthermore, emerging hardware technologies, particularly photon-counting detector CT (PCCT), hold tremendous promise. PCCT dramatically improves spatial resolution and utilizes ultra-high-resolution reconstruction algorithms that inherently suppress beam-hardening and blooming artifacts, which may soon mitigate the calcification-underestimation paradox identified in our conventional multidetector CT cohort [26,27].

### Limitations

Certain limitations must be acknowledged. First, as a retrospective single-center analysis, unmeasured residual confounding related to specific scanner-specific parameters, image quality metrics (e.g., signal-to-noise ratio), and proprietary post-processing algorithms cannot be entirely excluded. Second, the reliance on visual estimation for ICA, despite double-blinded reading, introduces inherent interobserver variability. Third, the dynamic factors contributing to artifacts, such as patient-specific heart rate variability during the scan, were not systematically quantified for each vessel segment. Future prospective multicenter registries incorporating quantitative coronary angiography (QCA) or intravascular ultrasound (IVUS) alongside advanced CT technologies are required to validate these findings and comprehensively map the calcification barrier.

## 5. Conclusions

CCTA underestimation of coronary stenosis is a nonlinear, vessel-specific phenomenon strongly influenced by calcification burden. Given the nonlinear risk distribution and the significant interactions with local coronary anatomy, attempting to establish a single, isolated diagnostic threshold for the total CAC score is methodologically flawed. The determination of clinical cutoffs requires further consensus; however, standard ordinal grading and vessel-specific assessments (e.g., GEE profiling) offer robust alternatives. Severe calcification significantly elevates the risk of under-calling stenosis specifically in the LAD, LCX, and RCA, while the left main artery remains largely protected. Clinicians should view heavy calcification not solely as a trigger for overestimation, but as a complex, territory-dependent risk modifier that demands rigorous scrutiny to prevent the critical underestimation of ischemic heart disease.

## Data Availability

The datasets used and analyzed during the current study are available from the corresponding author on reasonable request.
