# Supplementary material for: Nonlinear and Vessel-Specific Association Between Coronary Artery Calcium Burden and Coronary Computed Tomography Angiography Underestimation of Coronary Stenosis
Source: Rev Cardiovasc Med. 2026 Sep 8;27(9):52230. doi: 10.31083/RCM52230 (PMC13655512; doi:10.31083/RCM52230)
Supplement: Supplementary file 1 [file 2153-8174-27-9-52230-s1.zip › Supplementary Material/STROBE_Checklist.docx]

STROBE Statement—checklist of items that should be included in reports of observational studies

Page numbers refer to the final 15-page PDF of RCM52230 (published 26 August 2026).

|  | Item No. | Recommendation | Page  No. | Relevant text from manuscript |
| --- | --- | --- | --- | --- |
| **Title and abstract** | 1 | (*a*) Indicate the study’s design with a commonly used term in the title or the abstract | 1 | Abstract (Methods): “We retrospectively evaluated 673 patients without known coronary artery disease who underwent both CCTA and invasive coronary angiography (ICA) within 90 days, yielding 2692 vessel observations.” This indicates a retrospective design, but the abstract does not explicitly name it as an observational/cohort study; the full design is stated in Section 2.1 (page 2). |
|  |  | (*b*) Provide in the abstract an informative and balanced summary of what was done and what was found | 1 | The abstract reports the study aim, retrospective observational analysis (673 patients; 2692 vessel observations), outcome definition, RCS and GEE methods, key patient- and vessel-level results, sensitivity analysis, and a balanced conclusion emphasizing nonlinear and territory-dependent associations. |
| Introduction | | | |  |
| Background/rationale | 2 | Explain the scientific background and rationale for the investigation being reported | 1–2 | The Introduction describes the clinical role and limitations of CCTA, explains calcification-related blooming/beam-hardening artifacts and the clinical hazard of stenosis underestimation, and identifies gaps in prior patient-level analyses and assessment of vessel-specific heterogeneity. |
| Objectives | 3 | State specific objectives, including any prespecified hypotheses | 2 | “To address these knowledge gaps, this study investigated the nonlinear dose-response association between CAC burden and CCTA underestimation” and sought to characterize nonlinear risk trajectories and vessel-specific heterogeneity using GEE and RCS. |
| Methods | | | |  |
| Study design | 4 | Present key elements of study design early in the paper | 2 | “This retrospective, single-center observational study was conducted at Beijing Friendship Hospital between January 2019 and December 2023.” |
| Setting | 5 | Describe the setting, locations, and relevant dates, including periods of recruitment, exposure, follow-up, and data collection | 2 | The study was conducted at Beijing Friendship Hospital from January 2019 to December 2023. Eligible patients underwent ICA within three months (90 days) after CCTA. |
| Participants | 6 | (*a*) *Cohort study*—Give the eligibility criteria, and the sources and methods of selection of participants. Describe methods of follow-up  *Case-control study*—Give the eligibility criteria, and the sources and methods of case ascertainment and control selection. Give the rationale for the choice of cases and controls  *Cross-sectional study*—Give the eligibility criteria, and the sources and methods of selection of participants | 2–3 | Section 2.1 reports initial screening of 920 patients without prior confirmed CAD, the inclusion/exclusion criteria, and the final cohort of 673 patients (2692 vessel observations). Fig. 1 on page 3 summarizes participant selection and the analytical workflow. |
|  |  | (*b*) *Cohort study*—For matched studies, give matching criteria and number of exposed and unexposed  *Case-control study*—For matched studies, give matching criteria and the number of controls per case | N/A | Not applicable; no matching was used in this retrospective observational diagnostic-comparison study. |
| Variables | 7 | Clearly define all outcomes, exposures, predictors, potential confounders, and effect modifiers. Give diagnostic criteria, if applicable | 2–4 | Section 2.4 defines vessel-level and patient-level CCTA underestimation. Section 2.5 defines total and vessel-specific CAC exposure measures and categories. Section 2.6 lists covariates for the fully adjusted models, and page 4 specifies CAC × vessel interaction terms. |
| Data sources/ measurement | 8* | For each variable of interest, give sources of data and details of methods of assessment (measurement). Describe comparability of assessment methods if there is more than one group | 2–3 | CCTA acquisition and six-grade stenosis assessment are described in Section 2.2; ICA acquisition, blinded independent assessment, and the identical six-grade system are described in Section 2.3. CAC was measured on non-contrast ECG-gated CT using the Agatston method (≥130 HU; area ≥1 mm²) in Section 2.5. |
| Bias | 9 | Describe any efforts to address potential sources of bias | 2–3 | ICA stenosis was assessed by two independent readers blinded to CCTA findings, with discrepancies resolved by a third senior interventionalist. GEE with robust Huber-White sandwich standard errors addressed within-patient vessel clustering, and a prespecified sensitivity analysis excluded patients with any overestimation. |
| Study size | 10 | Explain how the study size was arrived at | 2 | The manuscript states that 920 patients were initially screened and 673 were included after applying the prespecified eligibility criteria. No separate power-based sample-size calculation is reported. |
| Quantitative variables | 11 | Explain how quantitative variables were handled in the analyses. If applicable, describe which groupings were chosen and why | 3 | CAC was analyzed continuously using restricted cubic splines with knots at the 10th, 50th, and 90th percentiles and the median CAC score as the reference, and categorically as 0, 1–99, 100–399, and ≥400. |
| Statistical methods | 12 | (*a*) Describe all statistical methods, including those used to control for confounding | 3–4; 6; 11 | Baseline comparisons used the Mann-Whitney U test and Chi-square/Fisher’s exact tests. Patient-level associations used RCS and three logistic models; vessel-level outcomes used GEE with exchangeable correlation and robust Huber-White standard errors. CAC × vessel interactions were tested. Tables 2–3 specify the Cochran-Armitage trend test, and the Fig. 7 caption specifies multivariable logistic regression for subgroup analyses. |
|  |  | (*b*) Describe any methods used to examine subgroups and interactions | 4; 9; 11 | Page 4 specifies CAC × vessel interaction terms. Section 3.8 on page 9 reports subgroup analyses, and the Fig. 7 caption on page 11 specifies multivariable logistic regression adjusted for Model 3 covariates with interaction p-values. The subgroup procedure is not otherwise detailed in Section 2.6. |
|  |  | (*c*) Explain how missing data were addressed | 3 | “Missing data were minimal (<1%) and handled using complete case analysis.” |
|  |  | (*d*) *Cohort study*—If applicable, explain how loss to follow-up was addressed  *Case-control study*—If applicable, explain how matching of cases and controls was addressed  *Cross-sectional study*—If applicable, describe analytical methods taking account of sampling strategy | N/A | Not applicable. The study did not involve longitudinal follow-up loss, case-control matching, or a complex sampling design requiring sampling-weight adjustment. |
|  |  | (*e*) Describe any sensitivity analyses | 3; 9; 11–12 | A prespecified sensitivity analysis excluded patients with overestimation in any vessel. Section 3.9 reports that 518 patients were excluded, leaving 155 patients (113 with underestimation), and the continuous RCS analysis was repeated; results are presented in Fig. 8 and Supplementary Figs. 1–2. |
| Results | | | | |
| Participants | 13* | (a) Report numbers of individuals at each stage of study—eg numbers potentially eligible, examined for eligibility, confirmed eligible, included in the study, completing follow-up, and analysed | 2–3 | A total of 920 patients were initially screened and 673 were included in the final analytical cohort, yielding 2692 vessel observations. Fig. 1 on page 3 displays cohort selection, exclusions, the final cohort, outcome definition, CAC assessment, and analytical workflow. |
|  |  | (b) Give reasons for non-participation at each stage | 3 | Fig. 1 reports 247 exclusions: interval >3 months between CCTA and ICA (n=53), missing imaging data or severe motion artifacts (n=92), and other prespecified exclusion criteria (n=102). |
|  |  | (c) Consider use of a flow diagram | 3 | Fig. 1 is the participant-flow diagram and summarizes screening, exclusions, the final analytical cohort, outcome definition, CAC assessment, and planned statistical analyses. |
| Descriptive data | 14* | (a) Give characteristics of study participants (eg demographic, clinical, social) and information on exposures and potential confounders | 4–5 | Section 3.2 summarizes baseline characteristics, and Table 1 on page 5 presents non-underestimation (n=284) and underestimation (n=389) groups, including demographic variables, vital signs, laboratory measures, comorbidities, total CAC score, and CAC categories. |
|  |  | (b) Indicate number of participants with missing data for each variable of interest | 3 | The manuscript states that missing data were minimal (<1%) and were handled by complete case analysis; the number missing for each individual variable is not reported separately. |
|  |  | (c) *Cohort study*—Summarise follow-up time (eg, average and total amount) | N/A | Not applicable; this diagnostic-comparison study did not include a longitudinal follow-up period to summarize. |
| Outcome data | 15* | *Cohort study*—Report numbers of outcome events or summary measures over time | N/A | Not applicable; outcomes were diagnostic discrepancies assessed from paired CCTA and ICA examinations rather than events accumulated over longitudinal follow-up. |
|  |  | *Case-control study—*Report numbers in each exposure category, or summary measures of exposure | N/A | Not applicable; this was not a case-control study. |
|  |  | *Cross-sectional study—*Report numbers of outcome events or summary measures | 4–6 | Any-vessel CCTA underestimation occurred in 389/673 patients (57.8%). Vessel-specific rates were LM 3.0%, LAD 27.9%, LCX 29.3%, and RCA 22.4%. Tables 2 and 3 report outcome counts and rates across patient-level and vessel-level CAC categories. |
| Main results | 16 | (*a*) Give unadjusted estimates and, if applicable, confounder-adjusted estimates and their precision (eg, 95% confidence interval). Make clear which confounders were adjusted for and why they were included | 5–10 | Patient-level RCS findings are reported in Section 3.5 and Fig. 3, including adjusted estimates and 95% CIs. Vessel-level RCS and GEE results are reported in Sections 3.6–3.7 and Figs. 4–6. In the fully adjusted vessel-level GEE model, patient-level CAC ≥400 versus 0 was associated with OR 2.01 (95% CI 1.20–3.36). Fully adjusted covariates are listed in Section 2.6. |
|  |  | (*b*) Report category boundaries when continuous variables were categorized | 3; 6 | CAC categories were defined as 0, 1–99, 100–399, and ≥400. For continuous RCS analyses, the median total CAC score (301) served as the reference value; Table 2 on page 6 reports results across the four CAC categories. |
|  |  | (*c*) If relevant, consider translating estimates of relative risk into absolute risk for a meaningful time period | N/A | Not applicable. The outcome was diagnostic underestimation rather than a time-to-event outcome, so relative estimates were not translated into absolute risks over a follow-up period. |
| Other analyses | 17 | Report other analyses done—eg analyses of subgroups and interactions, and sensitivity analyses | 9; 11–12 | Section 3.8 reports subgroup analyses, including a triglyceride interaction (p-interaction = 0.032), with stratum-specific estimates in Fig. 7. Section 3.9 and Fig. 8 report the sensitivity analysis excluding any-vessel overestimation (n=155), which retained a significant nonlinear association (p-overall = 0.012; p-nonlinear = 0.005). |
| Discussion | | | | |
| Key results | 18 | Summarise key results with reference to study objectives | 12–13 | The Discussion summarizes that CAC-associated CCTA underestimation is nonlinear and vessel-specific, that a single CAC cutoff is inadequate, and that integrated patient-level calcification burden and vessel-specific risk profiling better characterize diagnostic discordance. |
| Limitations | 19 | Discuss limitations of the study, taking into account sources of potential bias or imprecision. Discuss both direction and magnitude of any potential bias | 13 | The Limitations section identifies the retrospective single-center design and residual confounding, potential interobserver variability from visual ICA estimation, and unmeasured dynamic/image-quality factors. It recommends prospective multicenter validation with QCA/IVUS and advanced CT technologies; the magnitude of these potential biases is not quantified. |
| Interpretation | 20 | Give a cautious overall interpretation of results considering objectives, limitations, multiplicity of analyses, results from similar studies, and other relevant evidence | 12–13 | The Discussion interprets the nonlinear and vessel-specific findings in the context of calcification artifacts and prior evidence, emphasizes that RCS OR = 1 crossings are graphical reference points rather than validated diagnostic cutoffs, and recommends integrated CAC and territory-specific assessment rather than reliance on a single threshold. |
| Generalisability | 21 | Discuss the generalisability (external validity) of the study results | 13 | Generalisability is addressed in the Limitations by acknowledging the retrospective single-center design and calling for prospective multicenter registries to validate the findings. |
| Other information | |  | | |
| Funding | 22 | Give the source of funding and the role of the funders for the present study and, if applicable, for the original study on which the present article is based | 14 | “This work was supported by the National Natural Science Foundation of China (Grant No. 81600276).” The role of the funder is not separately described in the manuscript. |

*Give information separately for cases and controls in case-control studies and, if applicable, for exposed and unexposed groups in cohort and cross-sectional studies.

**Note:** An Explanation and Elaboration article discusses each checklist item and gives methodological background and published examples of transparent reporting. The STROBE checklist is best used in conjunction with this article (freely available on the Web sites of PLoS Medicine at http://www.plosmedicine.org/, Annals of Internal Medicine at http://www.annals.org/, and Epidemiology at http://www.epidem.com/). Information on the STROBE Initiative is available at www.strobe-statement.org.
