# Supplementary material for: Nonlinear and Vessel-Specific Association Between Coronary Artery Calcium Burden and Coronary Computed Tomography Angiography Underestimation of Coronary Stenosis
Source: Rev Cardiovasc Med. 2026 Sep 8;27(9):52230. doi: 10.31083/RCM52230 (PMC13655512; doi:10.31083/RCM52230)
Supplement: Supplementary file 1 [file 2153-8174-27-9-52230-s1.zip › Supplementary Material/Supplementary File.docx]

Supplementary Material

***S1. Standalone Diagnostic Performance of Continuous CAC Score***

**Given the highly non-linear risk trajectory confirmed in our primary analysis, traditional binary classification metrics are methodologically limited.** However, to empirically demonstrate the limitations of relying on a single absolute threshold, the isolated discriminative ability of the continuous total CAC score for predicting any-vessel underestimation was evaluated descriptively using standard receiver operating characteristic (ROC) analysis (Supplementary Fig. 1). The CAC score exhibited limited standalone diagnostic accuracy, yielding an area under the curve (AUC) of 0.551. Optimization utilizing the Youden index identified a single theoretical cutoff at CAC = 38.4. While this threshold provided high sensitivity (0.848), it resulted in poor specificity (0.271), indicating a high false-positive rate if used as a strict diagnostic boundary. **These findings empirically validate our decision to eschew a single mathematical cutoff in favor of multi-dimensional risk profiling in the primary manuscript.**

**Supplementary Fig. 1. Receiver operating characteristic (ROC) curve of total CAC score for predicting patient-level CCTA underestimation**. Notes: This ROC curve evaluates the standalone diagnostic discrimination of the continuous total CAC score for identifying patients with at least one underestimated coronary vessel. **Consistent with the non-linear nature of calcification artifacts,** the optimal cutoff point (38.4) determined mathematically using the maximum Youden index yields imbalanced sensitivity and specificity, **highlighting its unsuitability for direct clinical application.** The reference line (dashed) represents an AUC of 0.50. Abbreviations: AUC, area under the curve; CAC, coronary artery calcium; CCTA, coronary computed tomography angiography; ROC, receiver operating characteristic.

***S2. Diagnostic Performance in Sensitivity Analysis***

We performed a parallel ROC analysis within the sensitivity cohort **(excluding patients with any CCTA overestimation)** (Supplementary Fig. 2). In this subset, the discriminative capacity of the continuous CAC score remained poor, yielding an AUC of 0.481 (95% CI: 0.435–0.527) with a Youden-derived cutoff of 12.3. **The failure of the ROC model to demonstrate predictive utility in this pure underestimation phenotype—despite the highly significant non-linear association confirmed by RCS in the same cohort (see main text)—further reinforces the methodological inadequacy of linear thresholding.** These findings reinforce the conclusion that a single CAC threshold cannot accurately discriminate stenosis underestimation on its own. Instead, CAC burden functions primarily as an underlying, **non-linear** risk modifier that warrants increased visual scrutiny during CCTA interpretation.

**Supplementary Fig. 2. Sensitivity analysis of the ROC curve for continuous CAC score**. Notes: Receiver operating characteristic (ROC) curve assessing the diagnostic performance of the CAC score within the predefined sensitivity analysis cohort **(N = 155)**. The test yielded an AUC of 0.481 (95% CI: 0.435–0.527), indicating limited independent discriminative ability **when forced into a binary linear threshold**. The optimal theoretical cutoff was calculated at 12.3 based on the Youden index. Abbreviations: AUC, area under the curve; CAC, coronary artery calcium; CI, confidence interval; ROC, receiver operating characteristic.
